# A dual prokaryotic (*E*. *coli*) expression system (pdMAX)

**DOI:** 10.1371/journal.pone.0258553

**Published:** 2021-10-21

**Authors:** Manabu Murakami, Agnieszka M. Murakami, Shirou Itagaki

**Affiliations:** 1 Department of Pharmacology, Hirosaki University Graduate School of Medicine, Hirosaki, Japan; 2 Collaboration Center for Community and Industry, Sapporo Medical University, Sapporo, Japan; CSIR-Institute of Himalayan Bioresource Technology, INDIA

## Abstract

In this study, we introduced an efficient subcloning and expression system with two inducible prokaryotic expression promoters, arabinose and lac, in a single plasmid in *Escherichia coli*. The arabinose promoter unit allows for the expression of a FLAG-tagged protein, while the isopropyl-β-D-thiogalactoside (IPTG)-inducible unit allows for the expression of a Myc-tagged protein. An efficient subcloning (DNA insertion) system (iUnit) follows each promoter. The iUnit, based on a toxin that targets DNA topoisomerase of *E*. *coli*, allows for effective selection with arabinose or IPTG induction. With the dual promoter plasmid (pdMAX) system, expressed *lacZ* (β-galactosidase) activity was significantly decreased compared with the original solo expression system. Despite this disadvantage, we believe that the pdMAX system remains useful. A recombinant plasmid (pdMAX/ara/DsRed/IPTG/EGFP; pdMAX/DsRed/EGFP) with DsRed in the arabinose expression unit and EGFP in the IPTG expression unit showed fluorescent protein expression following additional low-temperature incubation. Thus, the novel pdMAX system allowed efficient subcloning of two different genes and can be used to induce and analyze the expression of two distinct genes. The proposed system can be applied to various types of prokaryotic gene expression analysis.

## Introduction

A number of bacterial expression plasmids are used for prokaryotic expression in *Escherichia coli (E*. *coli)*. At present, the use of different replication origins but compatible vectors is applied to analyze the simultaneous expression of two distinct proteins [1]. For example, the pET (pBR322 origin) vector can be used with the pRSET (pUC origin) vector (Novagen, Merck KGaA, Darmstadt, Germany). Due to differences in their plasmid copy numbers (the pRSET vector has high copy numbers, whereas pET does not), it is possible to obtain significantly more protein expression from pRSET than from pET in *E*. *coli*. A two-promoter system with the same T7 promoter has been established; however, the gene expression of each promoter cannot be independently regulated [2]. Therefore, a single-plasmid system with a differentially inducible promoter is required.

We previously established a solo expression (pgMAX) plasmid, a dual expression system with two (prokaryotic and mammalian) expression modes [3]. This novel system enabled efficient subcloning and gene expression in *E*. *coli*. Furthermore, this system enabled rapid construction of a mammalian expression vector with a simple deletion step and is referred to as a deletion (D)-type system.

In the present study, we established a dual promoter expression plasmid for use in *E*. *coli*. This novel dual expression (pdMAX) system has two inducible promoters, P_BAD_ for arabinose and the lac promoter for isopropyl-β-D-thiogalactoside (IPTG). To analyze the expression of inserted genes, we examined insertion of the *α*-peptide coding sequence of the *lacZ* gene, commonly referred to as the *α*-complementation assay, which enabled detection of *lacZ* activity via the formation of blue colonies [4]. Furthermore, we successfully induced two fluorescent protein genes (DsRed and enhanced green fluorescent protein, EGFP) via low-temperature incubation. The novel pdMAX system simplifies inducible prokaryotic gene expression analyses and will be useful for the expression analysis of different proteins in *E*. *coli*.

## Results and discussion

### Plasmid construction

**Fig 1:**
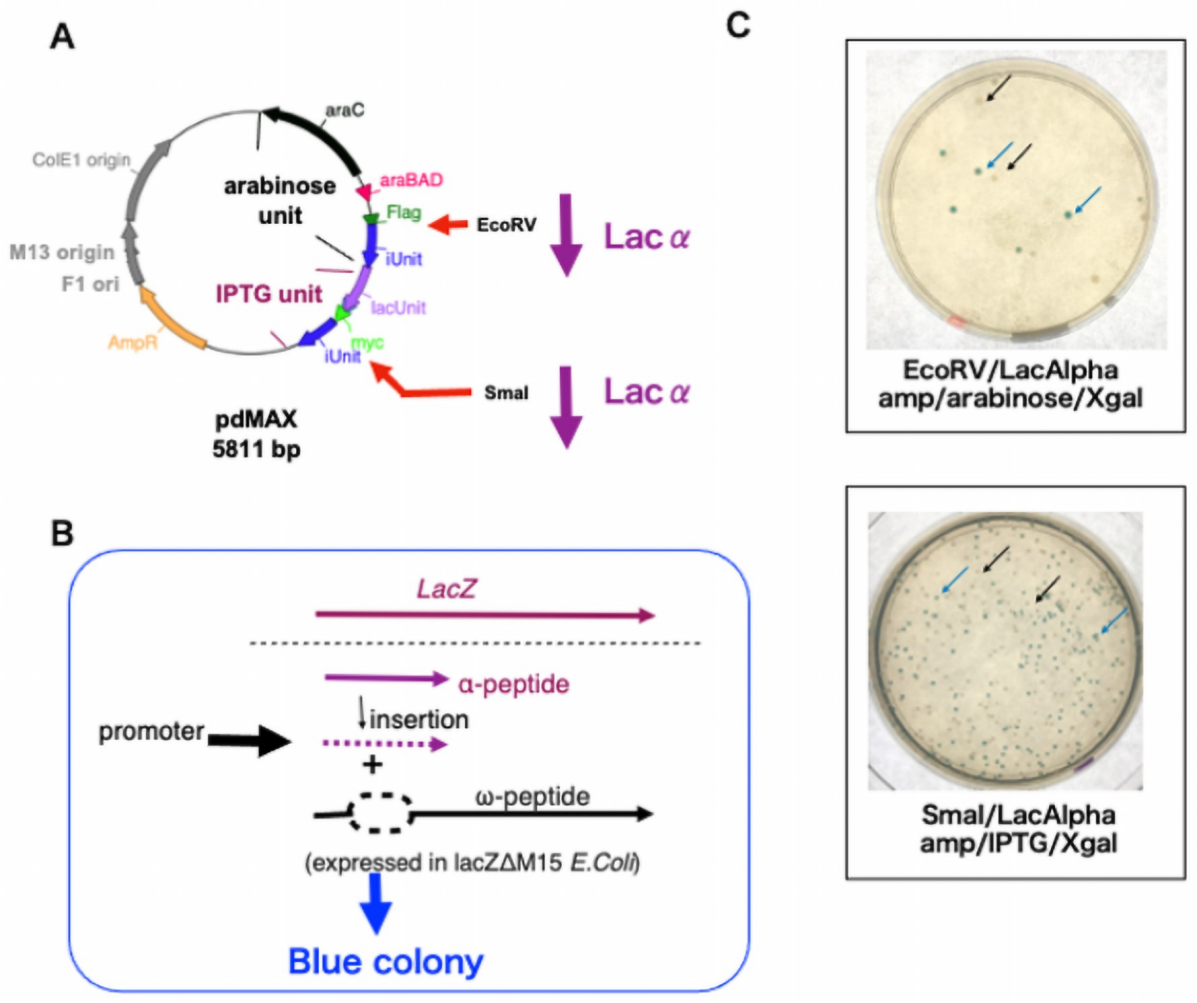
The pdMAX plasmid system. A. pdMAX/FLAG construct. The pdMAX system has two functional expression units: arabinose and IPTG. The subcloning (*Eco*RV and *Sma*I) sites for the α-peptide sequence (lac-α, purple arrow) are indicated. B. Scheme of the α-complementation assay. Polymerase chain reaction (PCR)-amplified blunt-ended α-peptide sequences were inserted into the cloning site. Following induction, the α-peptide was expressed along with the ω-peptide, resulting in α-complementation of *lacZ* (β-galactosidase). With active β-galactosidase, X-gal (a colorless analog of lactose) was cleaved to form an insoluble blue pigment. C. α-Complementation assay. PCR-amplified α-peptide was inserted into the *Eco*RV (arabinose expression unit, upper panel) or *Sma*I (IPTG expression unit, lower panel) site. The pdMAX construct harboring a lacZ α-peptide insertion in *Eco*RV was plated on plates containing amp, arabinose, and X-gal (*Eco*RV/LacAlpha). The pdMAX construct harboring a lacZ α-peptide insertion in *Sma*I was plated on plates containing amp, IPTG, and X-gal (*Sma*I/LacAlpha). Recombinant clones with lacZ α-peptide insertion in the sense direction resulted in blue colonies (blue arrows), whereas clones without the insert or insertion in the antisense direction resulted in white colonies (black arrows).

Fig 1A shows the plasmid map of pdMAX, which was based on that of pgMAX [3]. The novel pdMAX plasmid includes arabinose- and IPTG-inducible promoters. The arabinose expression unit featured *araC*, the *araBAD* promoter, the FLAG tag sequence, the *Eco*RV site for blunt-end cloning, and iUnit (Fig 1A); whereas the IPTG expression unit was composed of the lac promoter, the lac operator, the myc tag sequence, a *Sma*I site for blunt-end cloning, and an iUnit. Employment of the iUnit of CcdB (a toxin targeting the essential DNA gyrase of *E*. *coli* [5]) enables efficient selection of recombinant clones; bacteria with plasmids lacking inserts do not form colonies.

To confirm protein expression, the polymerase chain reaction (PCR)-amplified *α*-peptide sequence of the *lacZ (*β -galactosidase) gene was inserted between the blunt-end sites of *Eco*RV (in the arabinose expression unit) or *Sma*I (in the IPTG expression unit) of pdMAX. The host *E*. *coli* strain (XL-10) harbors the lacZ deletion mutation (lacZΔM15) that produces the ω-peptide [4]. Following ligation and transformation, the recombinant clones were plated on Luria–Bertani (LB) agar containing ampicillin (amp), X-gal, and arabinose (for arabinose induction) or IPTG (for lac operon induction). After 16 h, the numbers of blue (*α*-complementation) or white (antisense-directed ligation or no insertion of the DNA fragment) colonies were evaluated (Fig 1B).

Fig 1C shows representative images of α-complementation at the *Eco*RV (upper panel) and *Sma*I (lower panel) sites. Approximately 30–40% of colonies were blue, suggesting efficient insertion of the *α*-peptide gene sequence in the desired direction. When performing the α-complementation assay (insertion of a blunt-end DNA fragment of the α-peptide sequence), we did not perform alkaline phosphatase-mediated dephosphorylation, which is commonly employed to ensure that the vector does not re-circularize. The blunt ends generated via *Sma*I and *Eco*RV digestion are usually associated with a low rate of DNA insertion. The α-complementation assay revealed efficient exogenous DNA insertion when the iUnit method was employed (without this method, self-ligation of the vector plasmid was extensive). Also, the success rate of α-peptide ligation (yielding blue colonies) was good; the α-peptide was ligated in-frame in the correct (sense) direction.

### Dose-dependent induction of lacZ-α-peptide expression with arabinose or IPTG

In some cases, *α*-complementation can result in white or light blue colonies. Therefore, a secondary blue/white screening of the target colonies is required (S1 Fig) [6,7]. Secondary spot culture revealed decreased inducible gene expression in pdMAX, whereas the single-promoter pgMAX construct showed strong basal β-galactosidase activity, indicated by blue regions (S1 Fig, amp/Xgal, lane 3).

**Fig 2:**
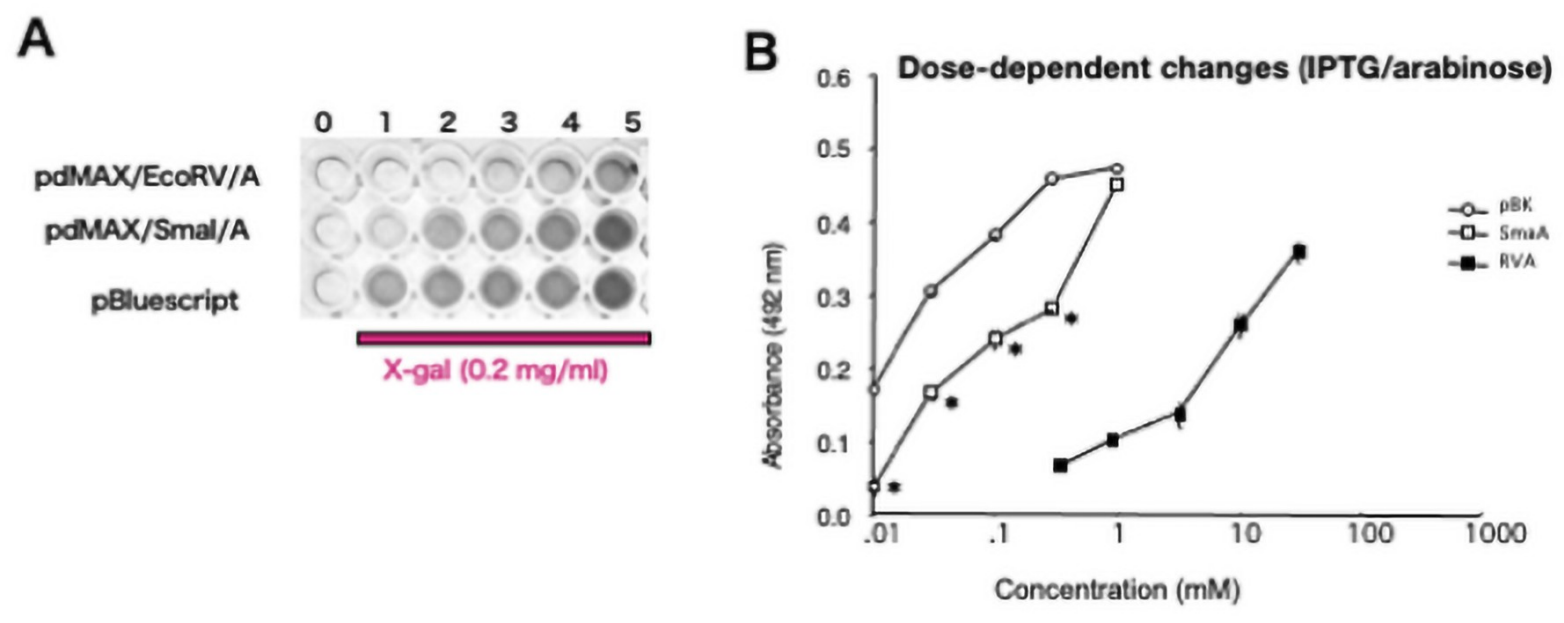
Dose-dependent induction of α-peptide. A. Representative image of *lacZ*-related blue *E*. *coli* clones. Lane 0, no X-gal, IPTG, or arabinose; lane 1, X-gal; lane 2, 0.01 mM IPTG or 0.3 mM arabinose; lane 2, 0.03 mM IPTG or 1 mM arabinose; lane 3, 0.1 mM IPTG or 3 mM arabinose; lane 4, 0.3 mM IPTG or 10 mM arabinose; and lane 5, 1 mM IPTG or 30 mM arabinose. B. Statistical analysis of absorbance of *lacZ*-related blue color. *Escherichia coli* with pBK (open circles) showed IPTG dose-dependent blue color absorbance. Recombinant clones of pdMAX/SmaI/α-peptide (SmaA) showed reduced but dose-dependent blue color absorbance following induction with IPTG (open squares). Recombinant clones of pdMAX/EcoRV/α-peptide (RVA) also showed arabinose concentration-dependent blue color absorbance (closed squares). N = 6. *p < 0.05 vs. pBK.

We explored the effects of inducer concentrations on *lacZ* expression. Fig 2A shows the pdMAX/EcoRV/A-associated, concentration-dependent color changes produced when the *α*-sequence was inserted into the arabinose expression unit (arabinose concentration: 3–30 mM), as well as the color changes produced when the *α*-sequence was inserted into the IPTG expression unit (IPTG 0.01–1.0 mM). The pBluescript plasmid (positive control), which harbors the *α*-sequence of the IPTG expression unit, exhibited significantly increased absorbance in the absence of IPTG induction (pBK). Presence of pBluescript significantly changed the absorbance of blue colonies in response to IPTG ((0.01–1.0 mM, Fig 2A and 2B). Arabinose induced a low level of the X-gal-associated blue color (pdMAX/EcoRV/; 3–30 mM). Statistical analysis showed concentration-dependent changes in the absorbance of blue colonies among the three constructs (Fig 2B). Compared with the single-promoter system (pBluescript), the dual-promoter system resulted in lower expression in response to IPTG. The EC_50_ of the single-promoter system was 0.03 mM; that of the dual-promoter system was 0.3 mM (thus ~10 times higher, assuming that a value of 0.475 reflected 100% efficacy at 492 nm; Fig 2B). Thus, the dual-promoter plasmid mediated less potent transgene expression compared with the single-promoter plasmid. Together, these results indicate that the dual inducible promoters (arabinose and IPTG) in pdMAX enabled concentration-dependent *lacZ*-*α*-peptide expression.

**Fig 3:**
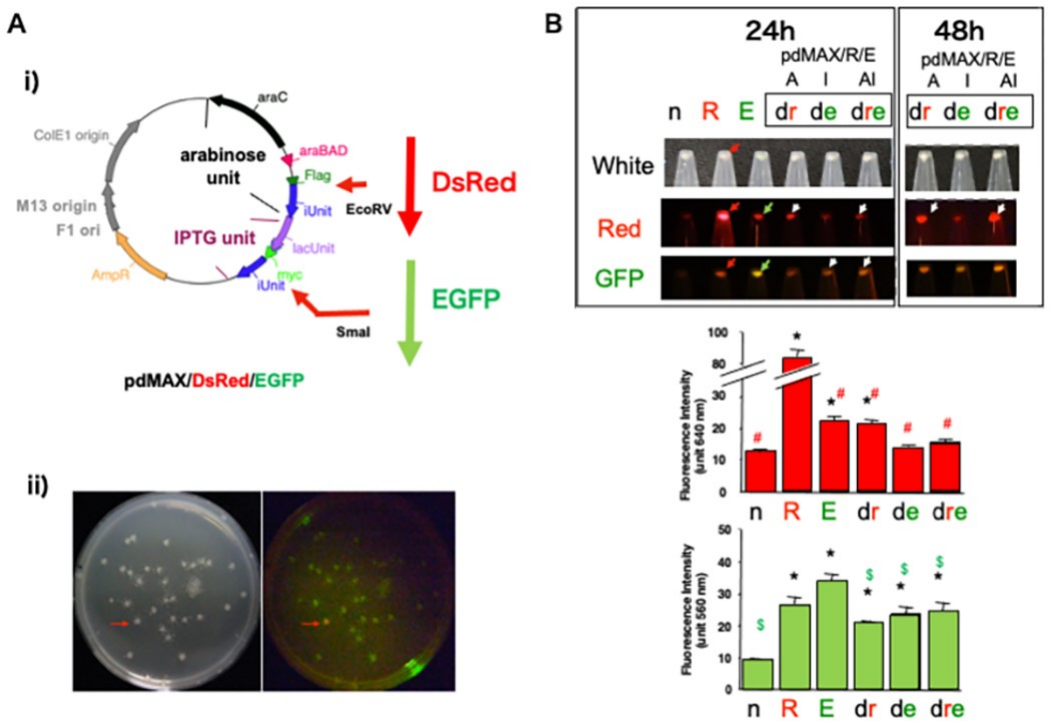
Fluorescent protein expression with pdMAX. A. Construction of pdMAX/DsRed/EGFP. i) Scheme of pdMAX and insertion sites of fluorescent protein genes. ii) Detection of red fluorescence after ligation of the DsRed gene into pdMAX. One colony showed red fluorescence (red arrows) after an additional 3 days of incubation under white light (left panel) and green light with Red set (right panel). B. Representative fluorescence in the pdMAX/DsRed/EGFP plasmid. Under white light, pgMAX/DsRed showed a faint red signal (red arrow). With the Red fluorescence filter (Red), pgMAX/DsRed showed strong fluorescence (red arrow), whereas pdMAX/DsRed/EGFP with arabinose induction showed a small fluorescence signal (white arrow, lanes dr and dre). With the GFP fluorescence filter (GFP), pgMAX/EGFP showed green fluorescence (green arrow), whereas pgMAX/DsRed showed red fluorescence (red arrow). The pdMAX/DsRed/EGFP construct with IPTG induction showed a small fluorescence signal (white arrow, lanes de and dre). The extended incubation times (24 or 48 h) are indicated. Bar graphs show the results of a statistical analysis of DsRed (upper) and GFP (lower) fluorescence intensity from the pdMAX/DsRed/EGFP (pdMAX/R/E) constructs under induction with arabinose (30 mM, lanes dr and dre) and IPTG (1.0 mM, lanes R, E, de, and dre) incubated at low temperature for 24 h). The pgMAX/DsRed construct showed strong red fluorescence at 640 nm (R). The pgMAX/EGFP construct showed the highest GFP fluorescence at 560 nm (E). Data are expressed as means ± standard error of the mean (SEM). For each group, n = 6. *p < 0.05 vs. negative control. ^#^p < 0.05 vs. pgMAX/DsRed. ^$^p < 0.05 vs. pgMAX/EGFP.

To further analyze protein expression in the pdMAX system, we inserted genes encoding a fluorescent protein (EGFP or DsRed). Insertion of DsRed or EGFP into the IPTG expression unit of the pdMAX plasmid resulted in marginal fluorescent protein expression (S1B 1 and S1B 2 Fig). Insertion of the EGFP gene into the *Sma*I site was efficient (success rate ~80%). However, pdMAX (the dual-promoter plasmid) resulted in lower EGFP protein expression compared with the single-promoter plasmid (pgMAX, S1B Fig). The DsRed gene was successfully inserted (success rate ~80%) into the *Eco*RV site of the arabinose expression unit, but only marginal DsRed fluorescence was observed after 16 h of incubation. The successful insertion of genes into either site facilitated iUnit expression, and the rates of blunt-end DNA fragment insertion were high. However, the insertion of DsRed or EGFP in the pgMAX plasmid, which has a single IPTG unit, resulted in high fluorescent protein expression (S1B, R and S1B E Fig), as reported previously [3]. The single-promoter plasmid (pgMAX) harboring the DsRed gene fluoresced much more strongly compared with the dual-promoter construct; the increases were 3.6- and 1.4-fold for the latter and former constructs, respectively (Fig 3B).

### Low-temperature incubation with the pdMAX system

For further analysis, we used the recombinant pdMAX plasmid, with DsRed in the arabinose expression unit and EGFP in the IPTG expression unit (pdMAX/DsRed/EGFP, Fig 3Ai). After ligation of the gene encoding the DsRed fluorescent protein, followed by 16 h of incubation at 37°C, no colony fluoresced. After 3 days of incubation at 4°C, only one of the 36 colonies evaluated exhibited DsRed fluorescence (Fig 3Aii, red arrow), probably attributable to the poor efficacy of the dual-promoter system. Also, *E*. *coli* exhibits a higher background fluorescence compared with mammalian cells. PCR screening revealed that one-third of colonies contained the desired insert oriented in the sense direction, but only one colony exhibited red fluorescence, suggesting that the dual-promoter system was ineffective.

Since the usual incubation protocol (16 h at 37°C) resulted in marginal expression of DsRed fluorescence using the dual-promoter system, we performed additional low-temperature incubation at 20°C for 24 h. After the additional 24-h incubation at a low temperature, fluorescent proteins with pdMAX/DsRed/EGFP were detected (Fig 3B, 24 h). Positive control clones with a single IPTG expression unit (pgMAX/DsRed and pgMAX/EGFP) showed strong fluorescent signals for DsRed (Fig 3B, middle panel, red arrow) and GFP (Fig 3B, bottom panel, green arrow). Both pgMAX/DsRed and pgMAX/EGFP showed overlapping fluorescent signals (Fig 3B; GFP, bottom panel, red arrow; Red, middle panel, green arrow). In contrast, the dual promoter pdMAX/DsRed/EGFP resulted in low but detectable expression of DsRed fluorescence upon arabinose induction (Fig 3B; red, middle panel, lanes dr and dre, white arrow). Induction with IPTG also resulted in increased EGFP fluorescence (Fig 3B; GFP, bottom panel, white arrow, lanes de and dre, white arrow). Statistical analysis after 24 h of low-temperature incubation revealed that pdMAX/DsRed/EGFP showed a detectable increase in DsRed fluorescence compared with that of the negative control (22.5 ± 1.4 and 13.1 ± 0.3 units for pdMAX/DsRed/EGFP and pdMAX, respectively; p < 0.05). In addition, pdMAX/DsRed/EGFP showed increased EGFP fluorescence (25.7 ± 1.3 and 10.2 ± 0.4 units for pdMAX/DsRed/EGFP and pdMAX, respectively; p < 0.05). An additional 24 h (48 h in total) of low-temperature incubation significantly increased the intensity of the DsRed fluorescent signal (Fig 3B; 48 h, Red, middle panel, white arrow), whereas GFP fluorescence increased to a smaller extent. *E*. *coli* exhibits non-negligible autofluorescence, attributable to endogenous flavins (emission wavelength 520–560 nm), NADH and NADPH (emission wavelengths 440–470 nm), and other molecules. Therefore, EGFP fluorescence may have been underestimated.

We next prepared a pdMAX/EGFP/DsRed construct (Fig 2A and S2 Fig). EGFP was inserted into the arabinose expression unit and DsRed into the IPTG expression unit (to yield pdMAX/EGFP/DsRed; S2A Fig). The construct evidenced both GFP and DsRed fluorescence after two days incubation at low temperature, indicating that both expression systems (arabinose and lac systems) facilitated expression of EGFP and DsRed (S2B Fig). Together, these findings reveal significant increases in DsRed and EFGP fluorescence with pdMAX/DsRed/EGFP, indicating the potential utility of the dual inducible promoter in a single-plasmid system.

In conclusion, we established an expression plasmid system for two different genes in *E*. *coli*. The pdMAX plasmid system allows highly efficient insertion of blunt-end DNA fragments such as PCR-amplified products, and inducible expression with arabinose or IPTG. The independent induction of two possibly interacting proteins would facilitate analysis of protein–protein interactions, including antigen–antibody interactions and ligand–receptor binding, and any inhibitory effect of the proteins on each other.

## Methods

All experimental procedures were approved by the Institutional Research Advisory Committee of the Hirosaki University School of Medicine (protocol 20T013).

### Plasmid construction

The novel pdMAXflag/myc originated from pgMAX [3]. The pBluescript plasmid was purchased from Agilent Technologies (Santa Clara, CA, USA). The pdMAX plasmid was constructed by serial PCR amplification and ligation. The araC gene and araBAD promoter were PCR-amplified from pBAD/His/lacZ (Invitrogen, Carlsbad, CA, USA). The lac promoter and inhibitory unit (iUnit) were PCR-amplified from pgMAX. The arabinose expression unit contains araC, the araBAD promoter, Kozak sequence, FLAG-tag sequence, *Eco*RV site, and iUnit. The IPTG expression unit contains the lac unit (lac promoter and lac operator), Kozak sequence, myc-tag sequence, *Sma*I site, and iUnit. The primers used in the construction of pdMAX are provided in S1 Table. PCR conditions using high-fidelity Pfu DNA polymerase (Agilent Technologies) were as follows: 35 cycles of denaturation at 94°C for 20 s, annealing at the calculated temperature for 30 s, and extension at 72°C for 30 s. Amplified PCR products were gel-purified with a gel extraction kit (Macherey-Nagel GmbH, Dueren, Germany). Protocols using the pdMAX system were based upon the former pgMAX system, which have been deposited in protocols.io (DOI dx.doi.org/10.17504/protocols.io.zq3f5yn).

### α-complementation assay (blue/white selection)

The host *E*. *coli* strain (XL-10) harbors the *lacZ* deletion mutant *lacZΔM15*) [4]. If the recombinant plasmid carries the *lacZ α*-sequence, which encodes the first 59 amino acids of β-galactosidase (the α-peptide), the ω- and α-peptides will be expressed together, resulting in a functional β-galactosidase enzyme [6,7]. On this basis, we inserted a PCR-amplified gene of the α-peptide sequence (corresponding to LQRRDWENPGVTQLNRLAAHPPFASWRNSEE) into blunt-end restriction enzyme sites (*Eco*RV or *Sma*I). If the α-peptide sequence is inserted in frame with the FLAG (in the arabinose expression unit) or myc (in the IPTG expression unit) sequences, and in the sense direction, the α-peptide is expressed following induction with arabinose or IPTG, resulting in the formation of blue colonies on X-gal-containing lysogeny broth (LB) plates.

For the *α*-complementation assay, a blunt-end DNA fragment (369 bp) of the *α*-peptide sequence of the *lacZ* gene was amplified using Pfu DNA polymerase with specific primers (AlphaFor, CAGGAAACAGCTATGAC; AlphaRev, CCATTCGCCATTCAGGCTGCGCAA) and pBluescript KS^-^ plasmid (Agilent Technologies) as a template. The PCR-amplified product was inserted at the *Eco*RV (arabinose promoter) or *Sma*I (IPTG promoter) site of pdMAX.

DNA ligation was performed using standard ligation techniques (Takara DNA Ligation kit ver. 2.1; Takara, Otsu, Japan). For transformation, XL10-Gold ultracompetent cells (Tet^r^ Δ(mcrA)183 Δ(mcrCB‒hsdSMR‒mrr)173 endA1 supE44 thi‒1 recA1 gyrA96 relA1 lac Hte [F’proAB lacI^q^ZΔM15 Tn10 (Tet^r^) Amy Cam^r^]; Agilent Technologies) were used. After 16 h of incubation on LB agar plates containing ampicillin (amp; 150 μg/mL), X-gal (1 mM), and IPTG (1 mM) or arabinose (10 mM), colonies were observed.

### Spot culture of recombinant clones

Confirmed by restriction analysis after small-prep DNA analysis, recombinant clones were diluted 100 times with LB medium and 2 μL of each clone was spotted on amp (with/without arabinose, IPTG, with/without X-gal)-containing plates. After 16 h at 37°C, colonies were observed.

### Evaluation of blue colonies (reflecting α-complementation)

To measure enzymatic activity of *lacZ*, *E*. *coli* cells with the recombinant plasmid were incubated in LB medium (0.5 mL) with X-gal (1.0 mM) on an orbital rocker at 37°C for 16 h with varying concentrations of IPTG (0.01–1 mM) or arabinose (0.3–30 mM). After the incubation period, each culture medium (0.3 mL) was transferred to a 96-well plate (0.35 cm^2^). The accumulation of blue-colored precipitates, indicating β-galactosidase activity, was evaluated at 490 nm (GENESYS 10S UV-Vis; Thermo Fisher Scientific, Waltham, MA, USA).

### Subcloning and fluorescent protein expression analysis

A blunt-end DsRed2 DNA fragment was amplified using Pfu DNA polymerase with DsRed2-specific oligo DNA (DsRed2for: AaaGCTAGCatgGCCTC CTCCGAGAAC GTCATCA; DsRed2rev: aaaGAATTCagatctcaggaacaggtggtg). A blunt-end EGFP DNA fragment was amplified using high-fidelity Pfu with EGFP-specific oligo DNA (EGFPfor: cccGCTAGCatgGTGAGCAAGGGCGAGGAG; EGFPrev: cccGGTACCGGCGGCGGTCACGAACTCCAG). The PCR-amplified fluorescent genes were inserted into the *Eco*RV site of the arabinose expression unit or the *Sma*I site of the IPTG expression unit of pdMAX.

To evaluate fluorescent protein expression, we incubated *E*. *coli* cells with the recombinant plasmid in LB medium (5.0 mL) on an orbital rocker at 37°C for 16 h with IPTG (1 mM) and/or arabinose (30 mM). After 16 h of incubation, *E*. *coli* cells were further incubated at 20°C for 24 h to examine protein expression. We transferred *E*. *coli* grown in liquid culture (0.5 mL) to 1.5-mL tubes and centrifuged the tubes at 70,000 × *g* for 1 min. The tube was inverted and fluorescence due to DsRed (excitation, 515 nm; emission, 60 nm) or EGFP (excitation, 450 nm; emission, 560 nm) was evaluated using ImageJ software (National Institute of Mental Health, Bethesda, MD, USA).

### Statistical analysis

Data are expressed as the means ± standard error of the mean. Prior to statistical analyses, data were analyzed with the Shapiro–Wilk test. After confirmation of a normal distribution, statistical differences were further determined by Student’s *t*-test. *p* < 0.05 was considered to indicate statistical significance.

## Supporting information

S1 Fig A. Spot culture and α-peptide induction. B. Insertion of fluorescent protein sequences into pgMAX and pdMAX.
(TIF)

S2 Fig A. A schematic of pdMAX/EGFP/DsRed and the insertion sites of the fluorescent protein genes. B. Representative fluorescence of the pdMAX/EGFP/DsRed plasmid.
(TIF)

S1 Table Oligo-DNAs used for pdMAX construction. (TIF)

S1 Dataset (TIF)

S1 File (DOCX)
