# Supplementary figures and images for: A dual prokaryotic (E. coli) expression system (pdMAX)
Source: PLoS One. 2021 Oct 21;16(10):e0258553. doi: 10.1371/journal.pone.0258553 (PMC8530331; doi:10.1371/journal.pone.0258553)

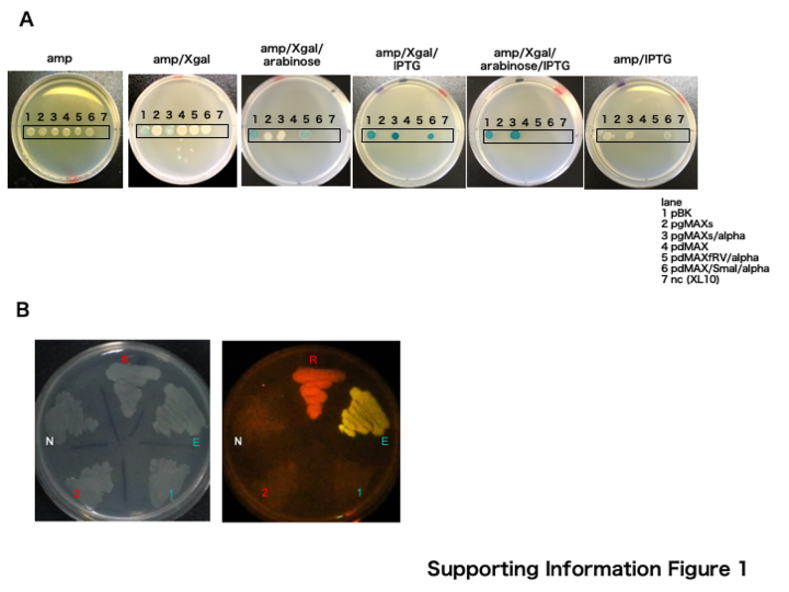

Supplement: S1 Fig — A. Spot culture and α-peptide induction. B. Insertion of fluorescent protein sequences into pgMAX and pdMAX. (TIF) [file pone.0258553.s001.tif]

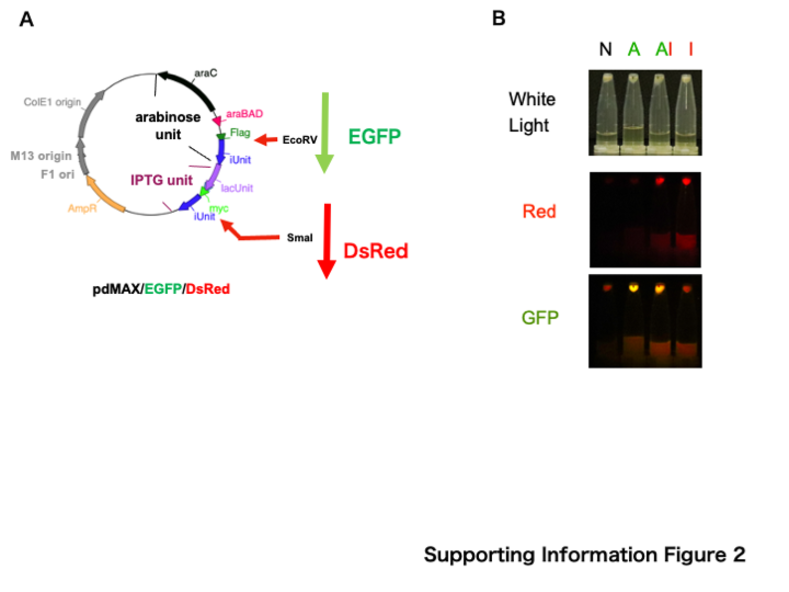

Supplement: S2 Fig — A. A schematic of pdMAX/EGFP/DsRed and the insertion sites of the fluorescent protein genes. B. Representative fluorescence of the pdMAX/EGFP/DsRed plasmid. (TIF) [file pone.0258553.s002.tif]

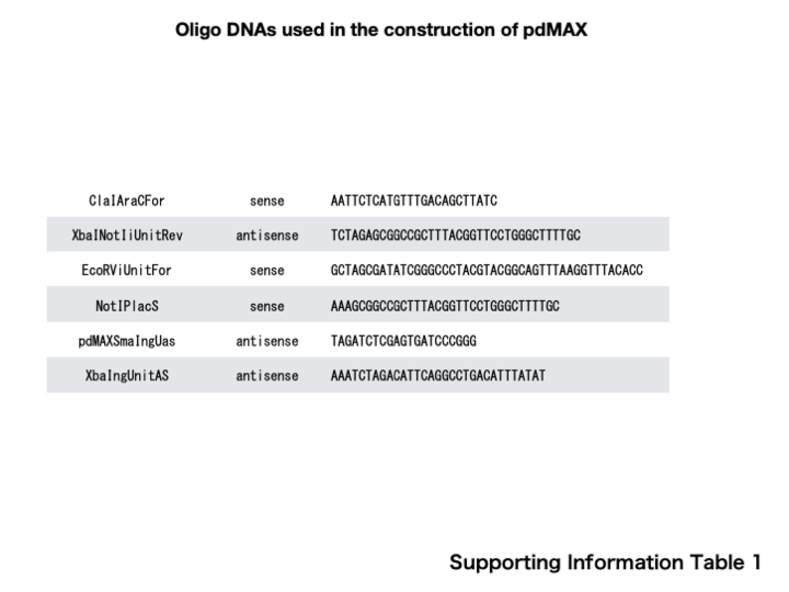

Supplement: S1 Table — (TIF) [file pone.0258553.s003.tif]

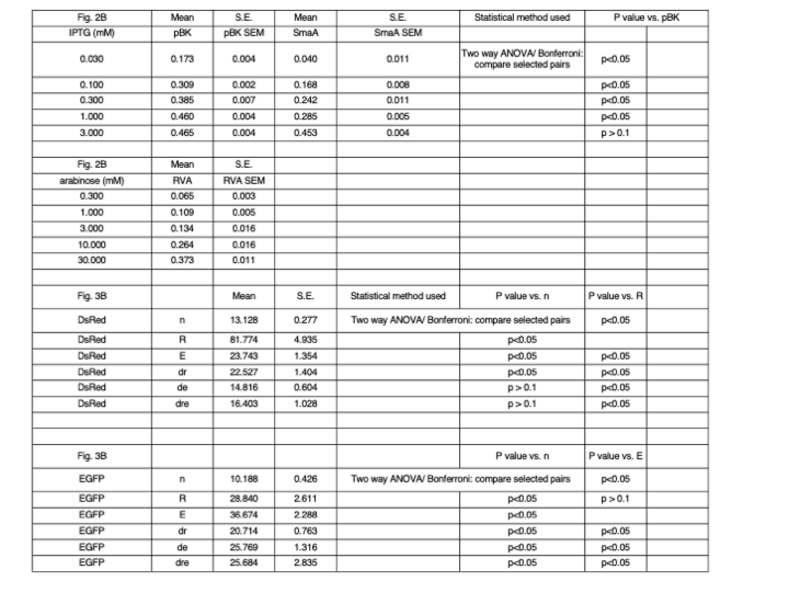

Supplement: S1 Dataset — (TIF) [file pone.0258553.s004.tif]
