# Supplementary material for: A dual prokaryotic (E. coli) expression system (pdMAX)
Source: PLoS One. 2021 Oct 21;16(10):e0258553. doi: 10.1371/journal.pone.0258553 (PMC8530331; doi:10.1371/journal.pone.0258553)
Supplement: S1 File — (DOCX) [file pone.0258553.s005.docx]

Supporting information

**Table S1.**

**Oligo-DNAs used for pdMAX construction**

**Figure S1.**

**A. Spot culture and α-peptide induction.**

Bacterial cultures hosting various plasmids were diluted (x100) and spotted (2 µL) onto LB plates. Lane 1, pBluescript (pBK); lane 2, pgMAX; lane 3, pgMAX/α-peptide; lane 4, pdMAX; lane 5, pdMAX with α-peptide-encoding DNA inserted into the *Eco*RV site of the arabinose expression unit); lane 6, pdMAX with α-peptide-encoding DNA inserted into the *Sma*I site of the IPTG expression unit); lane 7, negative control (nc; XL-10 cells). The LB plates were supplemented as follows: amp (50 µg/mL), X-gal (1 mM), arabinose (10 mM), and IPTG (1 mM). On amp/X-gal-containing plates, only pBluescript- and pgMAX/α-peptide-harboring bacteria formed blue colonies; bacteria harboring pdMAX with the α-peptide formed white colonies (lanes 5 and 6). On plates containing amp/X-gal/arabinose, bacteria with pdMAX containing the α-peptide inserted into the *Eco*RV site formed blue colonies (lane 5), whereas bacteria with pdMAX and pdMAX containing the α-peptide inserted into the *Sma*I site did not form colonies (lanes 4 and 6, respectively). On amp/X-gal/IPTG-containing plates, pdMAX containing the α-peptide at the *Sma*I site did not form colonies (lane 6).

**B. Insertion of fluorescent protein sequences into pgMAX and pdMAX.**

The fluorescence levels of bacteria hosting pgMAX/DsRed (R), pgMAX/EGFP (E), pdMAX/IPTG/EGFP (1), pdMAX/IPTG/DsRed (2), and pgMAX (N; negative control) growing on LB agar plates containing amp and IPTG. Colonies harboring pgMAX/DsRed (R), pgMAX/EGFP (E), pdMAX/IPTG/EGFP (1), pdMAX/IPTG/DsRed (2), and pgMAX (N) were observed under white light (upper panel). Fluorescence was observed under a blue light fitted with an orange filter (excitation wavelength, 450 nm; emission wavelength, 560 nm). The pdMAX plasmids containing EGFP (pdMAX/IPTG/EGFP, 1) and DsRed (pdMAX/IPTG/DsRed, 2) exhibited marginal fluorescence.

**Fig. S2.**

**A**. A schematic of pdMAX/EGFP/DsRed and the insertion sites of the fluorescent protein genes. The EGFP gene was inserted into the *Eco*RV site of araBAD. The DsRed gene was inserted into the *Sma*I site of the IPTG-inducible promoter.

**B.** Representative fluorescence of the pdMAX/EGFP/DsRed plasmid. After application of the DsRed fluorescence filter (Red), pdMAX/EGFP/DsRed exhibited red fluorescence after IPTG induction (lanes AI and I). After application of the GFP fluorescence filter (GFP), pdMAX/EGFP/DsRed exhibited GFP fluorescence after arabinose induction (lanes A and AI). Arabinose and IPTG induction triggered green fluorescence associated with slight red fluorescence (AI).

**Methods**

*Spot culture of recombinant clones*

α-Complementation can result in white or light blue colonies. Therefore, a secondary blue/white screening of the target colonies is required [7]. To confirm expression of the α-peptide or iUnits, inserted after each cloning site (*Eco*RV or *Sma*I), we examined secondary spot cultures of recombinant clones, which are modified *E*. *coli* cultures, as described by Zhang [7]. If α-complementation occurs on X-gal-containing plates, blue colonies form. If the iUnit is expressed, no colonies form [2]. Figure 2 shows typical images of different culture conditions. On amp-containing plates, pBluescript-containing clones showed white colonies (Supplementary Information Figure 1A, amp plate, lane 1). Other clones of pgMAX (IPTG-inducible iUnit-containing plasmid, lane 2), pgMAX with α-peptide (lane 3), pdMAX (lane 4), pdMAX with α-peptide at the *Eco*RV site (lane 5), and pdMAX with α-peptide at the *Sma*I site (lane 6) also formed white colonies, while XL-10 cells did not form any colonies (negative control, lane 7). Only pBluescript and pgMAX with α-peptide formed blue colonies on amp- and X-gal-containing plates amp/X-gal lanes 1 and 3), while pdMAX with the α-peptide inserted at the *Eco*RV or *Sma*I sites formed white colonies, indicating marginal expression of the α-peptide in both clones without gene induction (amp/X-gal, lanes 5 and 6). Arabinose induction resulted in blue colony formation in pBluescript and pdMAX with the *α*-peptide at the *Eco*RV site (amp/X-gal/arabinose, lanes 1 and 5), indicating induction of the *α*-peptide into the arabinose expression unit. Meanwhile, arabinose induction resulted in no colony formation with pdMAX and pdMAX with the *α*-peptide inserted at the *Sma*I site, indicating expression of iUnit in these transformants (lanes 4 and 6). IPTG induction showed blue colonies in pBluescript, pgMAX with the *α*-peptide, and pdMAX with the *α*-peptide at *Sma*I (amp/X-gal/IPTG, lanes 1, 3, and 6), indicating expression of the *α*-peptide and *α*-complementation. IPTG induction resulted in no colony formation in pgMAX, pdMAX, and pdMAX with the *α*-peptide at the *Eco*RV site (lanes 2, 4, and 5), indicating expression of iUnit. Taken together, spot culture analysis confirmed induction of arabinose and IPTG expression units. Interestingly, only pBluescript and pgMAX with the *α*-peptide showed blue colonies on X-gal-containing plates (Supplementary Information Figure 1A, amp/Xgal, lanes 1 and 3), while pdMAX with the *α*-peptide at *Eco*RV or *Sma*I resulted in white colonies (lanes 5 and 6), indicating low expression of the *α*-peptide in these two clones. Considering the sensitivity of the *lacZ* system, our data suggest that the pdMAX system results in low basal expression of the arabinose and IPTG expression units.

*Insertion of fluorescent protein sequences in pgMAX and pdMAX.*

The insertion of DsRed or EGFP into the IPTG expression unit of the original pgMAX plasmid, which features only a single such unit, resulted in strong expression of DsRed (R) and EGFP (E) fluorescent proteins (Supplemental Information Figure 1B). In contrast, the insertion of DsRed or EGFP in the IPTG unit of pdMAX (dual inducible plasmid) resulted in marginal fluorescent protein expression (Supplemental Information Figure 1B; 1, EGFP and 2, DsRed).

*Differential fluorescent protein expression in pdMAX*

The EGFP gene was inserted into the *Eco*RV site of the arabinose expression unit and the DsRed gene into the *Sma*I site of the IPTG expression unit. Fluorescence protein expression was evaluated following additional low-temperature incubation at 20^o^C for 24 h. Arabinose and IPTG induction successfully induced each fluorescent protein (Supplementary Information Figure 2B).
